# Supplementary material for: Combination Effects of Integrin-linked Kinase and Abelson Kinase Inhibition on Aberrant Mitosis and Cell Death in Glioblastoma Cells
Source: Biology (Basel). 2023 Jun 25;12(7):906. doi: 10.3390/biology12070906 (PMC10376030; doi:10.3390/biology12070906)
Supplement: Supplementary file 1 [file biology-12-00906-s001.zip › biology-2318258-supplementary.pdf]

Supplementary Figure S1

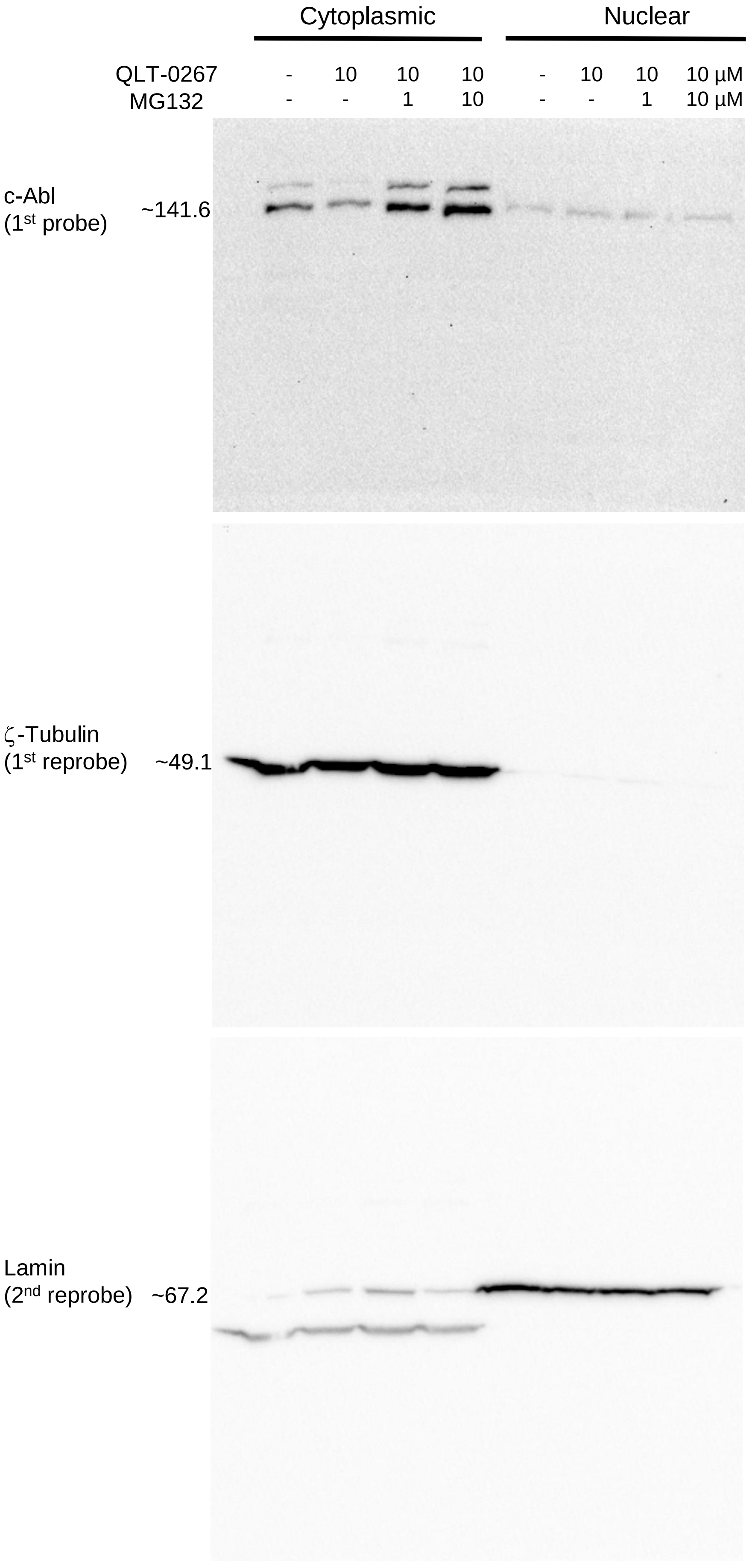

Supplementary Figure S1. The ILK Inhibitor QLT-0267 Decreases ABL in the Cytosolic Fraction and the Proteasome Inhibitor MG132 Rescues These Effects in T98G Cells. T98G glioblastoma cells were exposed to either DMSO or 10 µM QLT-0267 alone or together with 1 µM or 10 µM MG132 for 6 hours. Western blot analysis was performed following drug exposure, cell lysis and fractionation. Increasing amounts of MG132 in the presence of QLT-0267 rescued cytoplasmic ABL levels. ABL levels were determined using a rabbit antibody (Cell Signalling Cat #2862). The purity of cellular fractions was verified by reprobing the blots for tubulin, a primarily cytosolic protein (using a mouse α-tubulin antibody; Sigma Cat #T6199) or lamin, a primarily nuclear protein (using a rabbit α-lamin B1 antibody; Abcam Cat #ab16048). The additional lower weight molecular bands seen in the lamin Western blot are likely due to incomplete stripping of the α-tubulin antibody following the first reprobe.
